# Continuing the sequence? Towards an economic evaluation of whole genome sequencing for the diagnosis of rare diseases in Scotland

**DOI:** 10.1007/s12687-021-00541-4

**Published:** 2021-08-20

**Authors:** Michael Abbott, Lynda McKenzie, Blanca Viridiana Guizar Moran, Sebastian Heidenreich, Rodolfo Hernández, Lynne Hocking-Mennie, Caroline Clark, Joana Gomes, Anne Lampe, David Baty, Ruth McGowan, Zosia Miedzybrodzka, Mandy Ryan

**Affiliations:** 1 Health Economics Research Unit, University of Aberdeen, Aberdeen, UK; 2 Evidera Inc., London, UK; 3 Department of Medical Genetics, University of Aberdeen, Aberdeen, UK; 4 NHS Grampian Regional Genetics Service, Aberdeen Royal Infirmary, Aberdeen, UK; 5 South East Scotland Clinical Genetics Service, Western General Hospital, Edinburgh, UK; 6 NHS Tayside Regional Genetics Service, Ninewells Hospital, Dundee, UK; 7 South East Scotland Clinical Genetics Service, Queen Elizabeth University Hospital, Glasgow, UK

**Keywords:** Whole genome sequencing, Diagnostic odyssey, Costs and benefits, Economic evaluation, Valuation

## Abstract

Novel developments in genomic medicine may reduce the length of the diagnostic odyssey for patients with rare diseases. Health providers must thus decide whether to offer genome sequencing for the diagnosis of rare conditions in a routine clinical setting. We estimated the costs of singleton standard genetic testing and trio-based whole genome sequencing (WGS), in the context of the Scottish Genomes Partnership (SGP) study. We also explored what users value about genomic sequencing. Insights from the costing and value assessments will inform a subsequent economic evaluation of genomic medicine in Scotland. An average cost of £1,841 per singleton was estimated for the standard genetic testing pathway, with significant variability between phenotypes. WGS cost £6625 per family trio, but this estimate reflects the use of WGS during the SGP project and large cost savings may be realised if sequencing was scaled up. Patients and families valued (i) the chance of receiving a diagnosis (and the peace of mind and closure that brings); (ii) the information provided by WGS (including implications for family planning and secondary findings); and (iii) contributions to future research. Our costings will be updated to address limitations of the current study for incorporation in budget impact modelling and cost-effectiveness analysis (cost per diagnostic yield). Our insights into the benefits of WGS will guide the development of a discrete choice experiment valuation study. This will inform a user-perspective cost–benefit analysis of genome-wide sequencing, accounting for the broader non-health outcomes. Taken together, our research will inform the long-term strategic development of NHS Scotland clinical genetics testing services, and will be of benefit to others seeking to undertake similar evaluations in different contexts.

**Supplementary Information:**

The online version contains supplementary material available at 10.1007/s12687-021-00541-4.

## Introduction

Rare conditions collectively affect approximately 8% of the Scottish population (Scottish Government 2014). Many are severe and life threatening, with a profound impact upon the quality of life and wellbeing of the individual and their family. The typical journey to diagnosis involves a complex pathway of clinical and genetic tests, hospital appointments and considerable uncertainty for patients and families. This ‘diagnostic odyssey’ can take many years. Whilst Scottish patients wait an average of 4 years before receiving a diagnosis for their rare condition, many never receive a diagnosis (Scottish Government 2014). The diagnostic odyssey has a detrimental impact on health-related quality of life and well-being (Hilbert et al. 2013). Given around that 80% of rare conditions have a genetic origin, increasing access to genetic testing is a key component of global health policy decisions that aim to improve the lives of people with rare diseases and their families (Stark et al. 2019).

Novel developments in genomic medicine, such as whole genome sequencing (WGS) and whole exome sequencing (WES), may offer an opportunity to shorten the diagnostic odyssey for patients and families who live with unexplained rare conditions. The comprehensive sequence analysis of a person’s entire genome may increase the proportion of cases receiving a positive molecular diagnosis, enabling quicker diagnosis for rare diseases (Ontario Health 2020). In the UK, initiatives such as the 100,000 Genomes Project have delivered WGS using gene panel analysis to patients in a research context. Following their involvement in the 100,000 Genomes Project through the Scottish Genomes Partnership (SGP) (Scottish Genomes Partnership 2020), the Scottish Government announced a £4.2 million bridge funding package for NHS Scotland’s genetics services, in part to support ongoing evaluation of WGS and to inform future service delivery. This interim funding aims to support the continued momentum of genomics for the diagnosis of rare conditions in Scotland, acting as a bridge until there is sufficient evidence to inform a Scottish genomic testing strategy (NHS Scotland 2019).

Whilst there is a growing literature on the costs (Schwarze et al. 2018; Plöthner et al. 2017; Van Nimwegen et al. 2016; Tsiplova et al. 2017) and benefits (Chassagne et al. 2019; Marshall et al. 2016; Peyron et al. 2018; Regier et al. 2015) of WGS, to date, no studies have addressed this issue in a Scottish context. In research conducted alongside the Scottish bridge funding, and funded by the SGP and Chief Scientist Office of the Scottish Government, we are conducting an economic evaluation of WGS in Scotland (Chief Scientist Office 2019). Whilst the cost per QALY framework (Weymann et al. 2019; Lejeune and Amado 2021; Buchanan et al. 2021) is employed within Scotland to make recommendations for drugs (Scottish Medicines Consortium 2017) and technologies (Scottish Health Technologies Group 2017), decisions regarding the provision of genomic services currently lie with the National Services Division (NSD) of NHS Scotland. ‘V*alue for money*’ decisions are often being made on the basis of cost-effectiveness, with a focus on outcomes such as diagnostic yield, adverse events and hospital attendances, as well as budget impact considerations (National Services Scotland 2019, 2019). However, the recent report of the Scottish Scientific Advisory Council (SSAC) on the future of genomic medicine (SSAC 2019) identified the need to use both ‘standard and value-based health economic models to compare efficiency, cost-effectiveness and patient perspectives’.

This paper reports on research we have conducted to inform an economic evaluation of WGS in Scotland. At this time, we are not considering these findings with respect to diagnostic yield or in comparison with exome tests, nor undertaking formal cost-effectiveness or cost–benefit analysis. Instead, we seek to identify aspects of study design that must be considered to effectively evaluate genome-scale testing for clinical diagnosis. The rest of the paper is organised as follows. We first provide information on the context of our study, the Scottish Genomes Partnership (SGP). This is followed by the methods used to estimate the cost of singleton standard genetic testing and trio-based whole genome sequencing. We then present the qualitative methods used in an interview study designed to identify the benefits of whole genome sequencing. We then present our findings for each of component of the study, and draw our findings together in a discussion, highlighting future areas for research to ensure we can accurately assess and compare the costs and benefits of standard care and genomic sequencing for the diagnosis of rare disorders in Scotland.

## Study context: the Scottish Genomes Partnership

Our study participants are probands and parents from the SGP study, ‘*Scottish Participation in the 100,000 Genomes Project*’ (Scottish Genomes Partnership 2020). SGP is a pan-Scotland initiative designed to explore applications of genomics to solve clinical problems through academic, NHS clinical and industrial collaborations. Alignment of the SGP with the 100,000 Genomes Project is a policy aim of the Scottish Government (SG), supported through direct funding of associated research by the SG’s Chief Scientist Office (CSO) and the Medical Research Council (MRC). For research with NHS rare disease cohorts, the SGP developed a research collaboration with Genomics England for participation in the 100,000 Genomes Project to investigate the extent to which WGS with collaborative analysis could improve genetic testing and clinical follow-up for rare disease patients. ‘Collaborative analysis’ refers to the collaboration between the SGP and Genomics England, between Scotland’s four regional genetics centres, as well as between clinicians, scientists and universities. The SGP study operated under a distinct research protocol to the rest of the UK with some key differences. Of relevance here, Scottish samples were not sent to Genomics England for sequencing, but instead sequenced in Scotland with data then shared with Genomics England.

Eligibility criteria for the SGP study are shown in Online Resource 1. Briefly, probands and first-degree relatives were recruited via NHS Scotland’s regional genetics services. DNA samples were extracted by the regional genetics laboratories and sent to the University of Edinburgh for sequencing using a HiSeqX. De-identified clinical data and encrypted FASTQ data files were transferred to Genomics England for generation of Variant Call and BAM files plus bioinformatics analysis, trio-based analysis and storage. Results were shared with the regional genetics laboratories for interpretation, validation and clinical reporting through a browser. At the time of this study, interpretation and reporting were ongoing; however, most patients/families have now received a study report.

## Methods

### Costing the standard genetic testing pathway

There is uncertainty around the nature and cost of both the standard genetic testing pathway and WGS for the diagnosis of rare conditions. Costing studies vary in both their definition of the standard testing pathway and components of cost included when evaluating WGS, making comparisons difficult (Schwarze et al. 2018; Yuen et al. 2018; Plöthner et al. 2017; Tsiplova et al. 2017; Payne et al. 2018; Ontario Health 2020). Furthermore, whilst the cost of trio-based WGS for the diagnosis of rare conditions has been estimated in an English context (Schwarze et al. 2018), no study has estimated the costs of standard genetic testing or trio-based WGS in Scotland specifically. We aim to fill this gap.

We define the standard genetic testing pathway as the *second-line* genetic testing component of the diagnostic odyssey undertaken *after* any initial chromosomal microarray, FISH and/or karyotype for singleton probands. Although a full diagnostic odyssey for rare diseases typically involves a wider range of clinical, biochemical and metabolic tests, followed by chromosomal microarray, FISH and/or karyotype, we assume that WGS would replace the genetic testing ‘*sub-odyssey*’ including single gene testing, multiple gene panel testing and epigenetic testing.

**Fig. 1:**
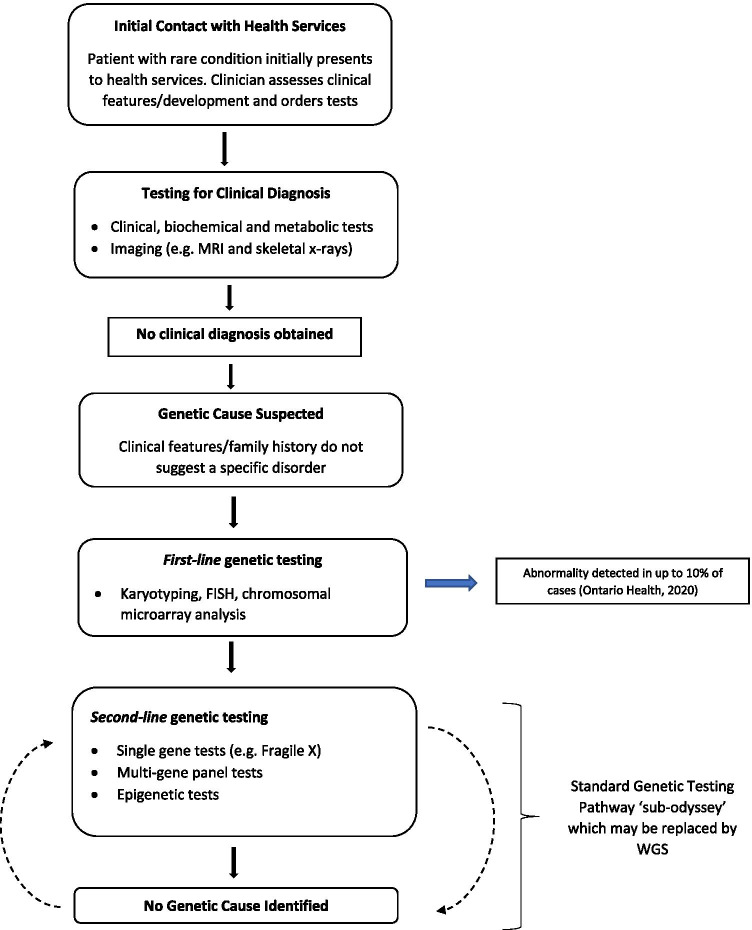
A typical diagnostic odyssey for a rare disease patient with a neurodevelopmental condition. This figure illustrates a typical ‘diagnostic odyssey’ for a rare disease patient with a neurodevelopmental condition. The iterative genetic testing ‘sub-odyssey’ is indicated by the black arrows. This portion of the overall diagnostic odyssey was costed in this paper, as it was deemed most likely to be replaced by WGS

An exemplar diagnostic odyssey for a singleton rare disease patient with a neurodevelopmental disorder, and the standard genetic testing pathway, or sub-odyssey, is presented in Fig. 1. The initial stage at which a rare disease patient presents to the health service depends on factors such as age and phenotype: adult-onset conditions may be more likely to be referred from primary care whilst conditions present from birth may be more likely to proceed immediately to specialist care. Additionally, the ending point of a diagnostic odyssey depends on the condition and what tests are available and can change over time as new tests are discovered.

The cost of the genetic testing sub-odyssey was estimated for each patient and each rare disease category. These categories were defined according to the Genomics England classification for rare diseases (Genomics England 2018) We extracted data on relevant prior testing histories from the SGP study participants’ WGS eligibility screening forms (see Online Resource 1), completed when patients were referred to Scotland’s four regional genetics centres. Eligibility screening forms were available for 393 probands. However, 134 probands were not costed due to missing information and/or unidentifiable genetic test names on eligibility screening forms (especially where handwritten). Data on testing histories for the remaining 259 probands did not include the date of the test, and therefore, it was not possible to estimate annualised costs for a proband’s standard testing pathway. All costs were estimated from an NHS Scotland perspective and reported in British Pounds (£) at 2018 prices.

For tests performed in Scotland, we attached unit costs provided by NHS Scotland’s genetics laboratories (reflecting likely costs to NHS Scotland). For tests conducted in laboratories in other parts of the UK, unit costs were sourced from these laboratories. This included several NHS England genetics laboratories. Additional information was sourced from the UK Genetics Testing Network. For tests where a unit cost was not available, costs were based on the number of amplicons or exons in the gene, following the GenU system for estimating resource use in laboratories for genetic tests (Norbury et al. 2016). Test costs increase with higher numbers of amplicons or exons as this reflects the number of days to report and therefore laboratory workload. An average cost per test was calculated for each of five ranges of number of amplicons or exons (1–10, 11–20, 21–30, 31–40 and 41 +). Further details of the sources for unit costs are shown in Online Resource 2.

To estimate the cost of associated hospital attendances (general outpatient and genetics clinic visits), unit costs were taken from NHS Reference costs (Reference Cost Collection, 2017–18). Due to unavailability of data on hospital attendances, our clinical advisors suggested an average of two outpatient appointments and one genetic outpatient appointment per patient.

### Costing trio-based whole genome sequencing

**Fig. 2:**
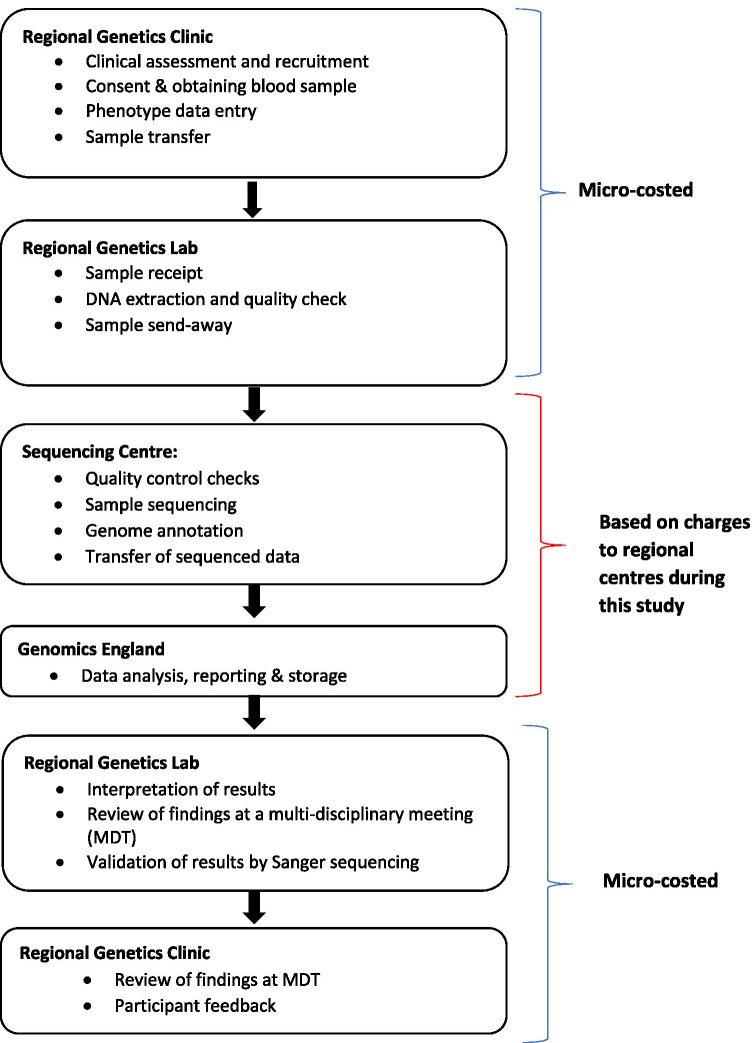
Figure 2 illustrates the whole genome sequencing pathway, as delivered in the Scottish Genomes Partnership’s involvement in the 100,000 Genomes Project. The parts of the WGS pathway which were micro-costed are indicated by the blue brackets, while the parts which were costed based on charges to the regional genetics centres are indicated by the red bracket

The WGS testing pathway reflects trio-based testing as per the SGP study protocol. A summary of the WGS pathway is illustrated in Fig. 2. Case eligibility discussion involved three or more consultant geneticists, typically covering five to seven cases in around one to 2 h, including preparation time. Consent was obtained by a genetics counsellor and phenotype data for all affected family members was entered into a database. Following review of stored DNA in the local diagnostic molecular genetic laboratory, 40% of probands did not require a new DNA sample. Entering phenotype data for all affected family members into the database was estimated to take between 15 min and 1 h. The regional genetics laboratory performed quality control checks before an aliquot of DNA was transferred into a 2D barcoded tube and sent to a central sequencing centre (Edinburgh Genomics) where sequencing was based on a service agreement for Human 30 × or 120 GB yield WGS, with PCR free libraries on the HiSeq X, returning FASTQ, BAM and VCF files. Coverage was calculated from good quality reads after removing overlapping bases and duplicated reads, after adaptor trimming, quality trimming and semi-aligned read clipping, and using well-mapped reads (mapQ > 10). Each FASTQ file contained at least 80 × 10^9^ bases with at least 95% genome covered at 15x or above.

An encrypted FASTQ data file created by the sequencing centre was then sent to GeL for data analysis after which a report is returned to NHS Scotland clinical scientists. Long-term (indefinite) data storage is provided by GeL. Each regional genetics laboratory received the analysis report for interpretation and validation, to confirm variants of clinical significance. Initially, results were interpreted by a clinical scientist in line with the American College of Medical Genetics (ACMG) guidelines (Richards et al. 2015) for determining pathogenicity, then discussed at a multi-disciplinary team (MDT) meeting. Where considered likely to be causative, findings were confirmed by Sanger sequencing. Our estimated cost per sample and per trio, sourced from the NHS Grampian laboratory, is based on Sanger sequencing on the original DNA sample and an assumed annual throughput of 100 samples (equivalent to 33 family trios).

Following laboratory validation, a report was issued to the original referring clinician and information relevant to the participant’s primary condition was fed back to families.

At the time of the study, WGS was not routinely practised in NHS Scotland and the process for reviewing results was not fully developed. We based our MDT meeting costs on the experience in the NHS Grampian genetics clinic during the SGP Project: It was assumed that 50% of WGS reports require discussion at an MDT meeting (based on the case mix seen in the NHS Grampian genetics clinic at that time) and that all probands attended follow-up.

Micro-costing, a technique, where detailed inputs of staff time, consumables and equipment requirements are estimated, was used to determine the resources used at NHS Scotland regional genetics laboratories and clinics (Raftery 2000). Staff time was estimated at salary grade mid-point with an allowance for clinic and laboratory overheads. Equipment costs are based on the annual equivalent costs for capital, plus allocated maintenance costs in 2018, adjusted using RPI inflation where required and discounted using 3.5% discount rate (Drummond et al. 2015) over specific lifetime of equipment. To calculate the capital cost per trio, the proportion of capital equipment time used for WGS was estimated based on diagnostic laboratory opinion. This was estimated to be < 1% for most capital charges, based on assumed annual throughput for laboratory processing of WGS samples.

For resource use outside of NHS Scotland regional genetics clinics and laboratories, micro-costing was not possible due to commercial confidentiality. Costings were thus based on charges to regional genetics centres, based on service agreements during the SGP study. This charge-based costing included sample sequencing by a central sequencing centre (Edinburgh Genomics) and data processing and storage by GeL.

As with standard genetic testing, all costs are estimated from an NHS Scotland perspective and reported in British Pounds (£) at 2018 prices.

### Identifying the benefits of whole genome sequencing in Scotland

Health economic evaluations of WGS and standard genetic testing have tended to define benefits in terms of diagnostic yield (the proportion of cases receiving a genetic diagnosis) (Weymann et al. 2019; Phillips et al. 2018; Schwarze et al. 2018; Smith et al. 2019; Zhang et al. 2019). More recently, however, there has been recognition of the relevance and importance of non-health outcomes and process factors to service users. This has led to a small but growing literature applying preference-based valuation methods, namely discrete choice experiments (DCEs) and willingness to pay (WTP) studies (Bennette et al. 2013; Buchanan et al. 2016; Chassagne et al. 2019; Goranitis et al. 2020; Lewis et al. 2018; Marshall et al. 2016; Peyron et al. 2018; Regier et al. 2015; Regier et al. 2018; Marshall et al. 2019; Weymann et al. 2018). This literature suggests that important factors extend beyond diagnostic yield, and include aspects such as waiting time for results, information provided and chance of improving health care provided and available treatments. However, no study has investigated the benefits of standard genetic testing or trio-based WGS to service users in NHS Scotland. We aim to fill this gap.

We explored what aspects of WGS patients and families with rare conditions in Scotland value. We conducted face-to-face interviews with a subset of SGP study probands and their families. Where possible, the methods used for this qualitative interview study were developed in accordance with the Consolidated Criteria for Reporting Qualitative Studies (COREQ) guidelines (Tong et al. 2007). The study was approved by Scotland A Research Ethics Committee (NHS Tayside) and NHS R&D (REC Ref. 16/NS/0137).

### Sample recruitment

We used a convenience sample of ten SGP probands and parents who were approached for consent and recruited following their appointment at the NHS Grampian regional genetics clinic. Eligible interview participants were English-speaking adults (over the age of 18) with a rare condition with residual unmet diagnostic need, or parents of a child with an undiagnosed rare condition. None of the participants had received WGS results prior to the interview. Following providing consent, individuals were invited to attend a single face-to-face interview. Participants were informed that the length of the interview would be between 30 and 45 min.

### Study process

Interviews were conducted in July 2018 at the Health Economics Research Unit (HERU), University of Aberdeen. A research fellow (BM) facilitated all interviews. Training in qualitative interview methods was provided by an experienced qualitative researcher at HERU. There was no relationship between the interviewer and the study participants prior to study commencement. Each interview was audio-recorded and transcribed by a professional third-party transcription provider. The interviews were conducted as ‘think aloud’ sessions, where participants were encouraged to verbalise their thought process when answering each question. This helps to ensure that respondents understand the questions whilst also providing valuable insight into the thought process behind each of their answers (Ryan et al. 2008; Rigby et al. 2020).

### Interview schedule

The interviewer followed a semi-structured script during the think aloud interviews. The interview schedule is available for reference in Online Resource 3. The schedule was informed by a systematic review of preference-based valuation in genomics and genetic testing (Weymann et al. 2019). To get respondents to think about the potential benefits of WGS, they were initially asked to rate five attributes of WGS in terms of their importance using a five-point scale whilst verbalising their thought process behind each rating. The themes were as follows: chance of receiving a diagnosis; waiting times for results; information for family members; information about other conditions; and contributions to research. These attributes were identified using expert clinical opinion and systematic review of the existing literature on preference-based valuation of WGS (Regier et al. 2009; Pélissier et al. 2016; Lewis et al. 2018; Peyron et al. 2018). Participants were also asked to comment on other factors important to them in the delivery of WGS. Throughout the interview, probing statements and prompts were used to encourage participants to engage with the ‘think aloud’ aspect of the interviews and to provide information about what is important to them in the delivery of WGS, and why. In addition to reminders to read and think aloud, the interviewer asked probing questions regarding the value of a diagnosis with and without available treatment.

To inform the levels of a cost attribute in our future DCE, we included a willingness to pay (WTP) question (Donaldson 1990). Individuals were asked directly how much they would be willing to pay for WGS where there is a chance (not a guarantee) that they may receive a genetic diagnosis; they can expect to wait between 6 months and 2 years for their results; health-related information would be provided to other family members if a genetic cause affects them; they can choose whether or not they receive information on secondary findings; and the research will benefit future healthcare for others with similar rare genetic conditions. Following participants’ initial response to the WTP question, the interviewer asked further probing questions about whether (and by how much) their WTP value would change if a diagnosis was guaranteed, and if treatment was available following a diagnosis.

### Analysis of qualitative data

An iterative thematic analysis approach was adopted to identify, extract and code relevant themes (Braun and Clarke 2014). One analyst (BM) identified relevant themes and extracted supporting quotations. A second analyst (MA) independently reviewed the interview transcripts and extracted supporting quotations. The wider research team discussed and refined the emergent themes and finalised the aspects of WGS which participants valued.

## Results

### Costing the standard genetic testing pathway

We identified a complex and varied range of testing histories. This included a range of initial imaging, biopsy, metabolic and biochemical investigations followed by first-line genetic testing (i.e. microarray, FISH and karyotype). Following these tests, the standard genetic testing pathway histories revealed a diagnostic sub-odyssey comprising multiple single gene tests, gene panels and in some cases, epigenetic testing. The total numbers of tests per patient in the diagnostic sub-odyssey ranged from one to sixteen (mean = 2.32; standard deviation (SD) = 2.08; median = 2; inter quartile range (IQR) = 2).

Cost estimates for the 259 probands are shown in Table 1. There was significant variability in the overall cost of the pre-WGS genetic testing pathway, with an estimated mean total testing cost of £1013 per singleton patient, and a median cost of £850. The distribution was right-skewed with a wide range, from £90 to £6784. Two outpatient appointments and one genetics clinic appointment gave an additional cost of £828. This results in a mean standard genetic testing pathway cost of £1841 per patient.

**Table 1:** Estimated costs of the standard genetic testing sub-odyssey

N = 259	
Mean (SD)	£1013.03 (£942.01)
Median	£850.00
Minimum; Maximum	£90.00; £6784.39
Hospital Attendances^a^	£828
**Total cost (mean + hospital attendances)**	**£1841**

^a^Assuming two general outpatient and one clinical genetics outpatient attendances. If there were the same number of general outpatient but twice as many clinical genetics visits, then the cost of hospital attendances would be £1064.78

**Fig. 3:**
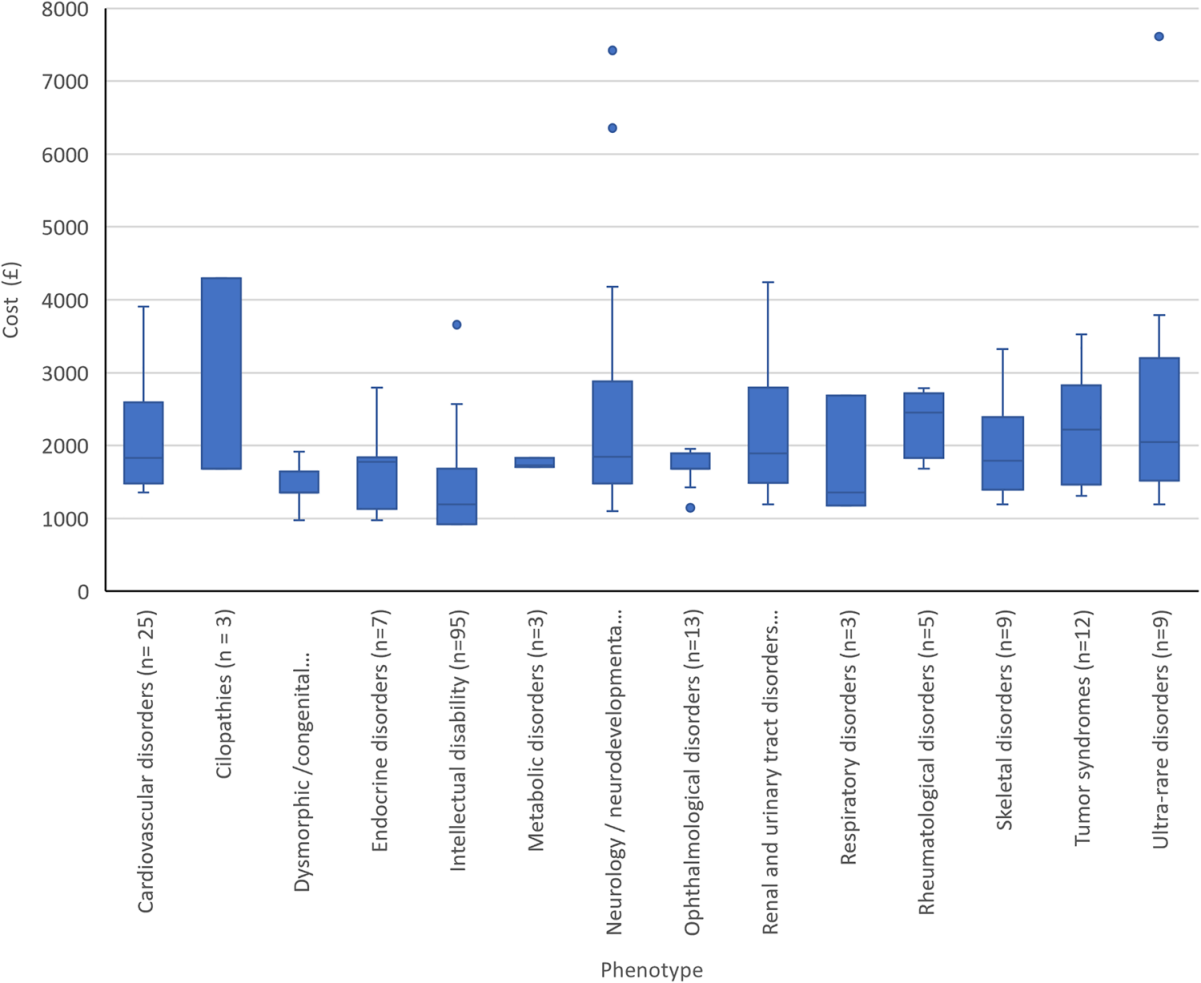
Costs of standard genetic testing by phenotype/rare disease category*.* Figure 3 illustrates the costs of the standard genetic testing pathway by phenotype category. The median cost of standard care for each phenotype category (for groups with *n* > 2) is shown by the horizontal line within each box. The outer whisker lines indicate the minimum and maximum costs for each phenotype category, excluding outliers. Outliers are shown as ‘dots’ and represent the observations with unusually high or low test costs in comparison to the median for that phenotype. Outliers were generally patients with large numbers of single gene tests and an expensive gene panel test. Note: The median cost of standard care for each category is shown by the horizontal line within each box. The outer whisker lines indicate the minimum and maximum costs for each category, excluding outliers. Outliers are shown as ‘dots’ and represent the observations with unusually high or low test costs in comparison to the median for that category. Outliers were patients with large numbers of single gene tests and an expensive gene panel test. ^1^Phenotype/rare disease categories defined by the Genomics England Classification system (Genomics England 2018)

The 259 patients fell into 19 rare disease categories. Results by rare disease category are shown in Fig. 3 for categories with three or more patients. The largest categories were intellectual disability (37%) and neurodevelopmental and neurological disorders without intellectual disability (21%). Five categories included 1–2 patients: gastroenterological disorders (*n* = 1); dermatological disorders (*n* = 1) and growth disorders (*n* = 1); genomic medicine service indications (*n* = 2) and hearing and ear disorders (*n* = 2).

Considerable cost variation was observed both between and within rare disease categories. For categories with more than two cases, the mean cost ranged from £1362 to £2701. Outliers were observed for two categories (neurological/neurodevelopmental and ultra-rare disorders), where the standard genetic testing pathway was estimated to cost around £7500; around three times greater than the mean cost for these patients. This was largely due to high numbers of single gene tests.

### Costing trio-based whole genome sequencing

Detailed costs for each key component, along with relevant underlying assumptions, are shown in Table 2. The total cost of WGS was £6625 per trio. All costs are reported in 2018 British Pounds.

**Table 2:** Whole genome sequencing costs

	Assumptions	Estimated cost (per trio)	% Total WGS cost
WGS	SGP Protocol (Trio)	£662,520	
Timing of WGS	After standard clinical, non-genetic, molecular genetic and cytogenetic testing		
Time horizon	1 year		
Screening and recruitment	Screening and recruitment undertaken by regional genetics centres	£132.63	2.00%
Consent and obtaining blood sample for DNA extraction	40% of probands did not require new sample to be taken. 75% probands requiring new sample needed a separate hospital visit for taking this sample	£96.42	1.46%
Phenotyping	Phenotype data entered into OpenClinica according to SGP protocol	£58.17	0.88%
Sample reception	Sample reception and book in at regional genetics laboratory	£8.80	0.13%
DNA extraction	Batch Size of 24	£186.88	2.82%
Sample send-away	Batch size 3 (Trio)	£30.00	0.45%
Sequencing of DNA	Cost charged to study by Edinburgh Genomics per Trio	£3,060.00	46.19%
Data analysis	As per 100,000 Genomes Protocol at GeL; cost charged to study by GeL	£1,684*	25.42%
Data storage	As per 100,000 Genomes Protocol by GeL; cost charged to study by GeL	£540.00	8.15%
Variant interpretation	Average of 4 variants per case (Trio-based analysis, exonic variants). No additional testing required to aid variant classification	£305.24	4.61%
Variant Validation	Potentially pathogenic variants confirmed by Sanger sequencing	£296.87	4.48%
MDT meeting to discuss findings	50% require MDT meeting. Time per case 5–20 min	£67.99	1.03%
Patient feedback	No data available on % trios who get feedback via genetics clinic visit. Costs assume all cases get feedback at clinic appointment	£158.20	2.39%

*Includes the cost of sending an encrypted FASTQ data file to Genomics England (GeL) for data analysis, including encrypted file transfer, generation of Variant Call and BAM files, bioinformatic analysis and return of results to NHS laboratories.

The estimated cost for screening for WGS eligibility was £132 per trio. Allowing for 40% of children not requiring a new DNA sample, estimated costs for consent and sample collection are £96 per trio. Phenotype data for all affected members was entered into a database, estimated to take between 15 min and 1 h, at an average cost of £58 per trio. The resource use by the regional genetics laboratory for DNA extraction was estimated at £187 per trio with an additional £30 per trio for sample send-away.

The largest component of the overall cost of providing WGS was the sequencing itself. Sequencing costs were based on the amount charged per sample (and pro rata per trio) by Edinburgh Genomics for the SGP WGS study. At the time of the study in 2018, this was £850 per sample, excluding VAT (£3,060 per trio, inclusive of VAT). This cost represented 46% of the overall cost of providing WGS.

The second-largest component of the estimated WGS cost was data analysis. The cost of an encrypted FASTQ data file being created by the sequencing centre and sent to Genomics England (GeL) for data analysis, including the generation of Variant Call and BAM files plus bioinformatic analysis, was estimated at £1,684 per trio, including VAT. The data analysis costs accounted for 25% of the overall WGS cost.

De-identified information from the GeL data analysis and report was stored securely at the GeL database at a cost of £540 per trio. Following data analysis at GeL, identified variants were interpreted at one of the four regional genetics laboratories, at a cost of £297 per trio. An estimate for the multi-disciplinary team (MDT) meeting was based on recent MDT experience in the NHS Grampian genetics clinic, and was £68 per trio. Finally, a follow-up appointment for discussion of test results, assuming all cases get follow-up, was £158 per trio.

### Identifying the Benefits of Whole Genome Sequencing

#### Sample information, genetic and demographic characteristics

Of the ten participants invited, nine consented to take part in the think aloud interviews. The sample consisted of six parents of children with rare conditions and three affected adults. The average length of time participants had been seeking a genetic diagnosis was 3.2 years for adult probands, and 3.6 years for children. However, this was highly variable, with a range of 6 months to 8 years and 1 to 6.5 years for adults and children, respectively. The age of onset of symptoms of the rare condition for adults in the sample ranged from 10 to 39 years, whilst the age of onset of children ranged from 5 months to 2 years. A wide range of testing was reported prior to WGS with two adults and six parents reporting genetic testing prior to their WGS referral: two adults and two parents reported operative procedures; one adult and five parents reported other non-genetic tests (e.g. MRI, CT, X-Ray) prior to enrolment in the SGP study.

#### Rating questions

All explored dimensions of benefit of WGS (chance of diagnosis, waiting time, information for family members, information about other conditions, contributions to research) were important to parents and affected adults. Four of the nine participants rated every attribute at the highest level of importance (5 = extremely important). When asked if any other factors were important to them about WGS, respondents consistently cited ‘having an answer’ and ‘closure’ as aspects of value. These factors were linked to the chance of receiving a diagnosis via WGS and reducing the uncertainty associated with the diagnostic odyssey.

#### Qualitative thematic analysis

Our thematic analysis revealed further insight into the value of WGS with respect to three aspects of value: a diagnosis; information provided; and contributions to research.

1. The value of a diagnosis

As expected, parents of children with rare conditions and affected adults consistently stated that they would value obtaining a diagnosis from WGS. The value of a diagnosis was generally centred around reducing the uncertainty associated with the diagnostic odyssey. Participants cited ‘*peace of mind*’, and ‘*closure*’ as reasons why they would value a diagnosis:

‘I think for your peace of mind it’s invaluable.’ – Parent 1

‘It would benefit [child] if he could get medication… they could maybe treat him… Benefit for mum and dad would be… just put closure? How could you explain that? Knowing a reason why gives us closure.’ – Parent 3

Notably, participants valued obtaining a diagnosis via WGS, even if there was no treatment available. When a probing question was asked about whether the participant would still value a diagnosis without any available treatment, several participants expressed that having a name for the condition would be valuable in itself:

‘But that would put your mind at rest a wee bit, do you know what I mean?’ – Parent 1

‘I just feel as a parent, if I knew what was wrong, I feel that I could have closure… So that would be my benefit as a parent.’ – Parent 3

‘I’d just want to know what’s wrong, whether you can solve it… you just don’t know what’s wrong with him, so it’s just the fact that you could put a label on it. It’s just that peace of mind that, okay, that’s what he’s got. It’s the unknown.’ – Parent 4

‘I think it’s the satisfaction of having an answer… Yeah, it’s satisfying curiosity. A bit of knowledge about the subject…I like to have an answer about things.’ – Parent 5

2. The value of the information provided by WGS

Further probing provided insight into two informational benefits of WGS. Firstly, participants valued information for family members regarding their chance of developing or passing on the condition. Secondly, patients and families valued receiving information regarding other health conditions unrelated to the rare condition (secondary findings).

‘When else would I get the opportunity to get that information? And when I have that information then I can maybe make a more informed decision myself about telling who in the family and so on, so I’m going to put extremely important.’ – Adult 1

3. Contributions to research and benefits beyond the individual

Both parents of affected children and affected adults recognised that, whilst there is a chance of diagnosis from WGS, there may not necessarily be available treatments or improvements in quality of life following the identification of a pathogenic genetic variant. Furthermore, participants were aware that a diagnosis via WGS is not a guarantee. Nevertheless, the possibility of benefiting future patients and contributing to the genomic evidence base was cited as valuable:

‘Because that’s why I’m here today. I hope to help others as well.’ – Adult 1

‘Extremely important because other people need help too.’ – Adult 3

‘Well, it seems like… this is not really for his benefit particularly. There’s a chance it could benefit him in some way, but realistically, it’s more to kind of go towards research and kind of… preventative future sort of things.’ – Parent 5

#### Willingness to pay for whole genome sequencing

Seven of the nine participants provided a WTP value for WGS, ranging from £200 to £5000 (Table 3). The two participants who did not provide a value indicated that they would pay for the test, but could not quantify how much they would be willing to pay.

**Table 3:** Willingness to pay values

Participant	Willingness to pay
A1	£200
A2	£2000
A3	£5000
P1	*Would pay but can’t quantify*
P2	£500
P3	£5000
P4	*Willing to pay for WGS if child’s quality of life improved*
P5	£2000
P6	£1000

*A* affected adult; *P* parent of affected child.

WTP probing questions provided further insight into what users of the service valued. Responses depended on the features of WGS described in the survey, particularly the chance of receiving a diagnosis and the possibility of improvement in quality of life. One respondent stated that they would be willing to pay much more for WGS if a genetic diagnosis was guaranteed. Similarly, one respondent stated that they would be willing to pay much more if there was a treatment available following a genetic diagnosis:

‘The price of something less for [child] to worry about is invaluable.’ – Parent 1

‘If I was to get a guaranteed result, I would pay whatever I needed to pay to get a result if it would help professionals make life better for [child], we’d pay anything.’ – Parent 3

‘I thought if there was a chance that there was going to be some sort of treatment from it, to help his condition, I think it would be, obviously, much more likely to spend money on…’ – Parent 5

‘If I knew we were definitely going to get an absolute result and there was something that might help us or more importantly help my daughter, what price would you put on that? It’s a really difficult question to answer.’ – Parent 6

## Discussion

In this exploratory study, we estimated the cost of standard (singleton) genetic testing and trio-based WGS according to the SGP study protocol. We also conducted qualitative research with SGP participants to inform the development of a DCE. Below we summarise our findings, as well as areas identified for further investigation.

### Assessing the costs of standard genetic testing

Standard genetic testing histories revealed a diagnostic ‘sub-odyssey’ comprising a complex range of single gene, gene panel and some epigenetic tests, with considerable heterogeneity between phenotypes. The mean cost of the standard genetic testing pathway, including hospital attendances, was £1841, with a range from £90 to £6784. We found significant variability in both the length and scope of the diagnostic odyssey. Outliers were generally cases with a large number of single gene tests or, in one case, a particularly costly gene panel test. Given that the consortium structure of genetics in Scotland is designed to minimise differences in the care patients receive, regional differences in how healthcare is accessed or delivered are unlikely to explain variations. We did not remove outliers since these cases are expected to occur in a rare disease population.

Our costing exercise has a number of shortcomings. Our costs were based on the prior testing histories of a sample of ‘difficult-to-diagnose’ patients, according to the SGP study eligibility criteria. These criteria limit the generalisability of our estimated costs beyond this patient group. Furthermore, our estimated costs are for singleton probands, and do not account for the cost of confirmatory and/or wider family testing. In cases where a genetic variant is identified via standard testing, confirmatory testing and wider family testing would typically take place. However, the SGP study eligibility criteria required that patients test negative on all prior genetic tests. Our clinical advice therefore suggested that the vast majority of standard genetic testing would be conducted on a singleton basis in this sample. As a result, any additional costs which might be incurred by other family members would be minimal in this context. It is likely that, in other contexts, where eligibility criteria do not require negative results on all standard genetic testing, confirmatory and wider family testing would result in higher costs.

Given the sample size, cost estimates for some phenotype subgroups lack precision. Our assumed number of hospital attendances may be an underestimate (given that patients often receive a series of tests over many years). Data on prior testing histories was based on eligibility screening forms, which only required sufficient information on prior genetic testing to assess eligibility for the SGP study. Different clinicians may supply varying levels of detail. As such, our costs may be an underestimate. Genetic testing histories in approximately one-third of screening forms could not be fully identified due to missing information, or where forms were handwritten and the name(s) of some genetic test(s) were unclear. We excluded these cases as full cost information could not be estimated for all tests. Furthermore, various sources were used to identify unit costs for standard genetic testing. Where possible, unit costs provided by Scottish genetics laboratories were used. However, in cases where Scottish unit costs were unavailable, UK unit costs were used. Where no UK unit cost data was available, costs were estimated based on the number of amplicons in the gene or gene panel. Combining these disparate sources of unit cost data may limit the consistency of our cost estimates. Additionally, we did not conduct a formal statistical analysis to quantify uncertainty in point estimates. Finally, due to data limitations, we could not annualise costs, and we thus do not know if higher standard care costs were due to longer diagnostic odysseys, more intensive care or both.

Future work will address the limitations of our costing estimates by extracting data from patients’ medical notes. More specifically, we will collect detailed data on SGP study probands’ prior genetic testing histories and relevant hospital attendances (including the year/month). Data will also be collected on patients’ genomic test report (positive, negative, variants of unknown significance), any parallel testing and additional testing for clarification. Details of any follow-up action taken resulting from the outcome of the genomic test will also be collected. This will allow us to more precisely characterise the genetic testing component of the diagnostic odyssey on an individual and annualised level. We will characterise the uncertainty around point estimates for the costs of the standard care pathway by conducting probabilistic sensitivity analysis.

### Assessing the costs of whole genomic sequencing

Trio-based WGS was estimated to cost £6625 per trio, reflecting WGS as per the SGP study protocol and early experience of WGS. Whilst the costing in this paper was limited to the practice in one of four Scottish genetics centres, communication with the other three Scottish genetics centres indicates that our estimates are generalisable across Scotland. However, we will extend our WGS micro-costing from one genetics centre in Scotland to all four regional genetics centres.

The costs of WGS estimated in this study should be interpreted in the context of the assumptions made, as well as in the context of the SGP study in Scotland. Varying the assumptions made regarding the WGS process will inevitably impact the estimated cost. At the time of the study, WGS interpretation was still bedding within NHS Scotland laboratories and clinics and the process for reviewing results was not fully developed. The costs associated with this element are expected to reduce as the process evolves. For example, MDT meeting formats tended still to be ‘atypical’ and on a learning curve as they continued to improve efficiency in terms of numbers/grades of staff required. The key driver of cost was sample sequencing, data analysis and data storage. Approximately 46% (£3060) of the total WGS cost was accrued from the sequencing itself, with a further 25% (£1684) at the data analysis stage. A reduction in these costs will significantly impact the total cost of providing WGS. Furthermore, bioinformatics and storage costs were high during the 100,000 Genomes Project, due to the upfront costs of developing the infrastructure from scratch. WGS is likely to be less expensive if delivered at scale (rather than in a research context). Similarly, advances in analytical methods that enable accurate variant filtering for singletons would reduce the cost of WGS testing, by reducing the need to sequence the DNA of family members (Boycott et al. 2013). Classification of variants is likely to become more automated and less labour intensive in the future, although any impact on the overall cost of WGS is uncertain.

Data were not available to consider the long-term implications on the diagnostic odyssey beyond the elements of the genetic testing pathway potentially replaced by WGS. The long-term and downstream cost implications of WGS are important to consider going forward, e.g. costs of confirmatory testing, data reanalysis, changes to patient management, incidental findings and reproductive decisions (Schofield et al. 2019; Doble et al 2020). However, this is beyond the scope of our study.

In this study, the comparator to standard genetic testing was restricted to WGS. Other novel developments in genomic medicine, such as whole exome sequencing (WES), which was delivered to patients in the Deciphering Developmental Disorders (DDD) study in Scotland (Firth and Wright 2011), may also offer a cost-effective alternative to standard care. Given that trio-based WES is being offered on an interim basis in a diagnostic context within NHS Scotland, we will also micro-cost WES in the same detail as WGS.

### Identifying the benefits of whole genome sequencing

Our think aloud interviews provide qualitative evidence that patients and families with undiagnosed rare conditions value a broad range of health and non-health outcomes associated with WGS. This provides support for the need to go beyond clinical measures of benefit such as diagnostic yield when assessing the value of genome-scale sequencing from a user perspective. For example, several participants cited ‘*peace of mind*’ and ‘*closure*’ as valuable aspects of WGS. These aspects of value were interpreted as being related to reducing the stress and uncertainty of the diagnostic odyssey. Additionally, patients and families recognise that, even if they do not receive a diagnosis immediately, their genome sequence data may benefit others in the future. Our WTP results provide further evidence of the value of WGS beyond diagnostic yield alone.

Our rating exercise found that all aspects of WGS (chance of diagnosis, waiting time for results, information provided by the test and contributions to research) were important to patients and families. It is possible that framing effects influenced this finding, with all aspects presented positively, e.g. chance of receiving a diagnosis and the possibility of contributing to research and helping others with undiagnosed rare conditions. However, this finding is more likely a consequence of the shortcoming of rating questions, where individuals do not have to make trade-offs between aspects of care (Cleland et al. 2018). A planned DCE will force individuals to make trade-offs between different aspects of care.

The main limitation of our think aloud study is the sample size, with nine respondents (six parents of affected children and three affected adults). Given that our findings are consistent with other qualitative studies, in similar contexts, we are confident that we have captured what is important to users of genomic testing in Scotland (Pollard et al. 2021; Mackley et al. 2018; Lewis et al. 2020; Goranitis et al. 2020; McCarthy et al. 2020). Nonetheless, future developmental work will explore this further. Our Project Advisory Group includes the Scottish Policy and Engagement Manager for Genetic Alliance UK; we will use this group to further explore what is important to users. Following this, we will develop a DCE which will be sent to all families affected by rare disorders who participated in (i) the SGP study collaborating with the 100,000 Genomes Project (excluding those who have participated in our developmental work) and (ii) the Deciphering Developmental Disorders (DDD) study. Our DCE results will be incorporated into a user-perspective cost–benefit analysis of WGS and WES vs standard care. This will allow us to value the broader benefits of genome-wide sequencing which are important to patients and families with rare conditions, such as the provision of information.

## Conclusions

Trio-based WGS is a costly investigation in comparison to the current standard genetic testing pathway in Scotland. However, an increase in diagnostic yield and additional value may justify the additional cost. Greater clarity is needed on both the costs and benefits of genome-wide sequencing to inform Scottish Government policy and its funding in clinical practice. This paper adds to the economic evidence base on assessing the costs and benefits of WGS for the diagnosis of rare conditions. Our exploratory costing study will guide a detailed costing of the diagnostic odyssey, with access to a larger sample and patient case notes and the update of our WGS costing to reflect changes in service delivery and costs since 2018. These updated costs will feed into a budget impact analysis, a cost-effectiveness analysis (cost per diagnostic yield) and a user perspective cost–benefit analysis (taking account of all factors important to patients and their families). Along with further developmental work, our qualitative think aloud findings will guide the development of a discrete choice experiment. This DCE will be sent to a large sample of patients and families with rare conditions in Scotland. Stated preference data will then be used in our user-perspective cost–benefit analysis, to account for the wide range of health and non-health outcomes which are important to patients and families. Taken together, our research will inform the long-term strategic development of NHS Scotland clinical genetics testing services and will be of benefit to others seeking to undertake similar evaluations in different contexts.

## Supplementary Information

Below is the link to the electronic supplementary material.

Supplementary file1 (DOCX 3723 KB)

Supplementary file2 (DOCX 23 KB)

Supplementary file3 (DOCX 97 KB)

## Data Availability

The authors may provide additional details of the study data on request.
