# Supplementary material for: Continuing the sequence? Towards an economic evaluation of whole genome sequencing for the diagnosis of rare diseases in Scotland
Source: J Community Genet. 2021 Aug 20;13(5):487–501. doi: 10.1007/s12687-021-00541-4 (PMC9530076; doi:10.1007/s12687-021-00541-4)

**Online Resource 1: Eligibility Criteria for the SGP study and** Eligibility Screening form for Participants in the Scottish Genomes Project Whole Genome Sequencing Study (copy of the form used by NHS Grampian)

**SGP Study Eligibility Criteria**

| **Inclusion criteria:** |
| --- |
| Rare disease with residual unmet diagnostic need  (Eligibility criteria as defined for the 100,000 Genomes Project (Genomics England, 2018)) |
| Access to appropriate family structures (ideally parent-offspring trio), with suspected Mendelian dominant, recessive, or X-Linked conditions |
| Multiple unrelated families with evidence of linkage to the same chromosomal region(s). |
| Families with suspected Mendelian conditions with one or more affected individuals in a single generation (likely to be due to de novo mutations). |
| Individuals with extreme forms of common disorders (e.g. particularly early onset or unusually severe forms) |
| Evidence of core phenotypes compatible with that disease, with evidence of previous genetic testing. |
| Particular conditions that have undergone a defined gene panel test or other tests to exclude known genetic mutations. |
| For the optional questionnaire development session, individuals must already have consented to take part in the SGP Study. |
| **Exclusion criteria:** |
| No evidence of an inherited phenotype or rare disease. |
| Prior identification of a known causative gene variant in an affected family member. |
| The absence of valid consent, or unwillingness to give consent or participate in all aspects of the Project (this excludes opt-out options for feedback to participants). |
| Inadequate phenotyping. |

^1^ *Genomics England, (2018) About the rare disease programme. Accessed January 2019. Available at:* [*https://www.genomicsengland.co.uk/about-genomics-england/the-100000-genomes-project/information-for-gmc-staff/rare-disease-documents/*](https://www.genomicsengland.co.uk/about-genomics-england/the-100000-genomes-project/information-for-gmc-staff/rare-disease-documents/)


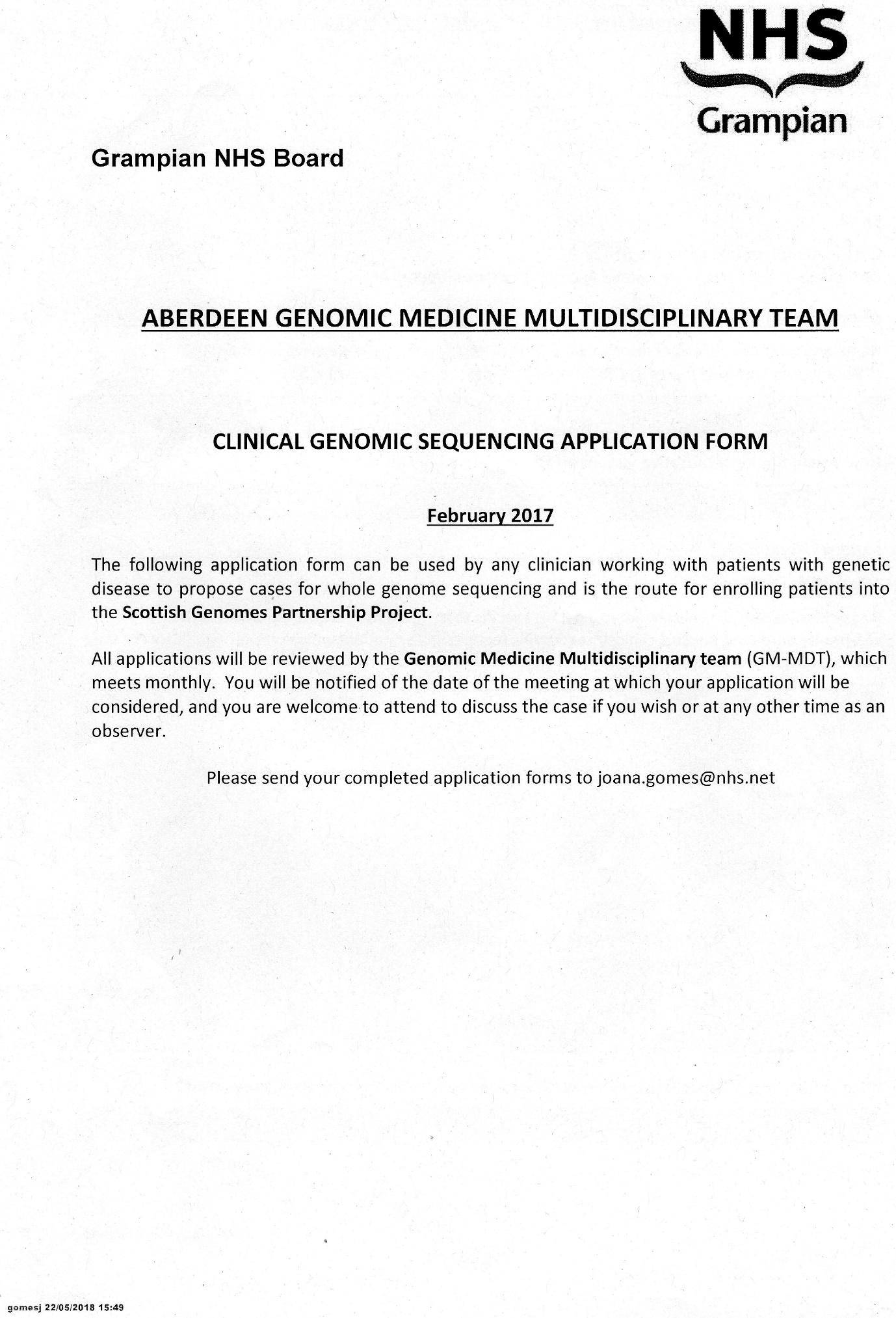


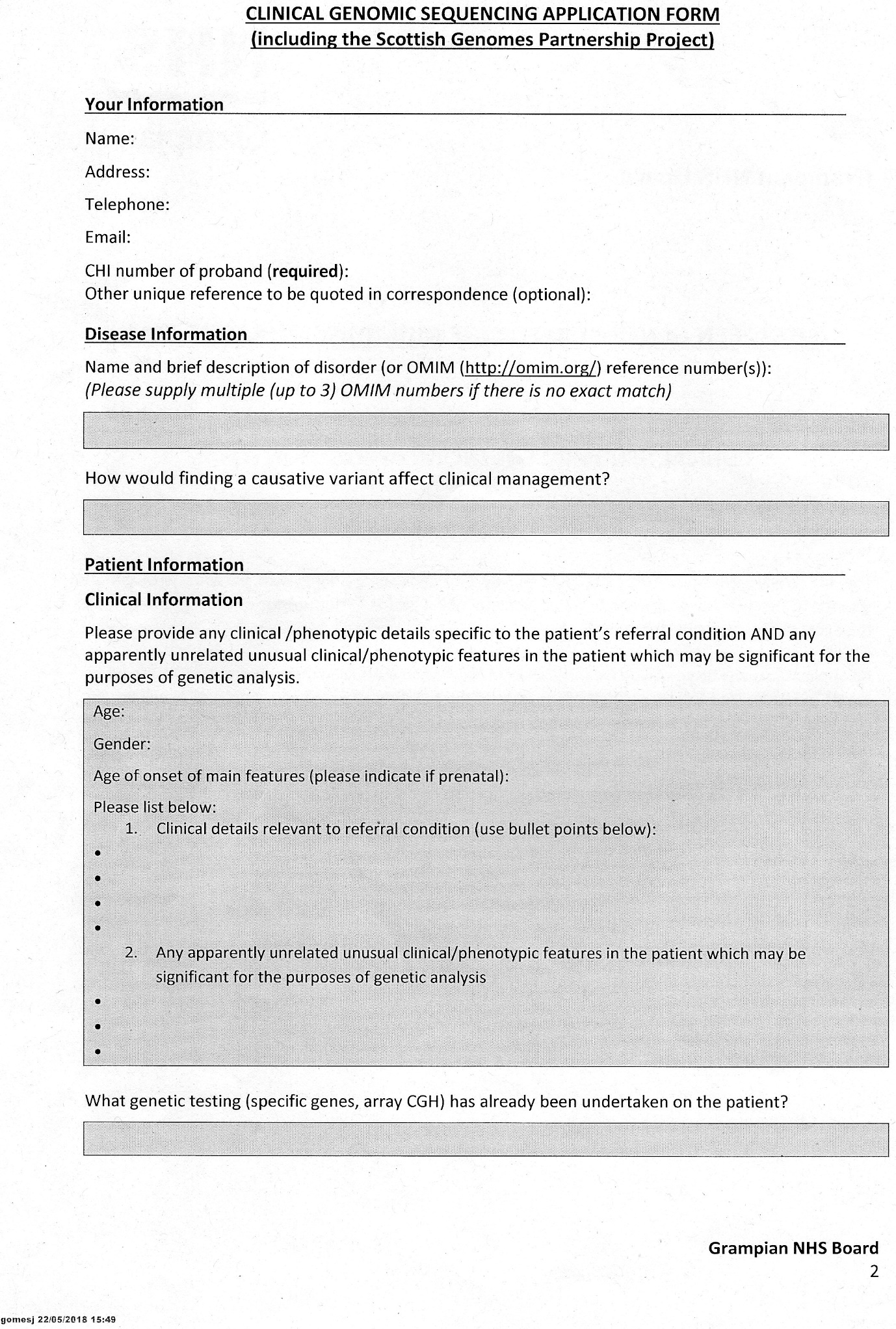


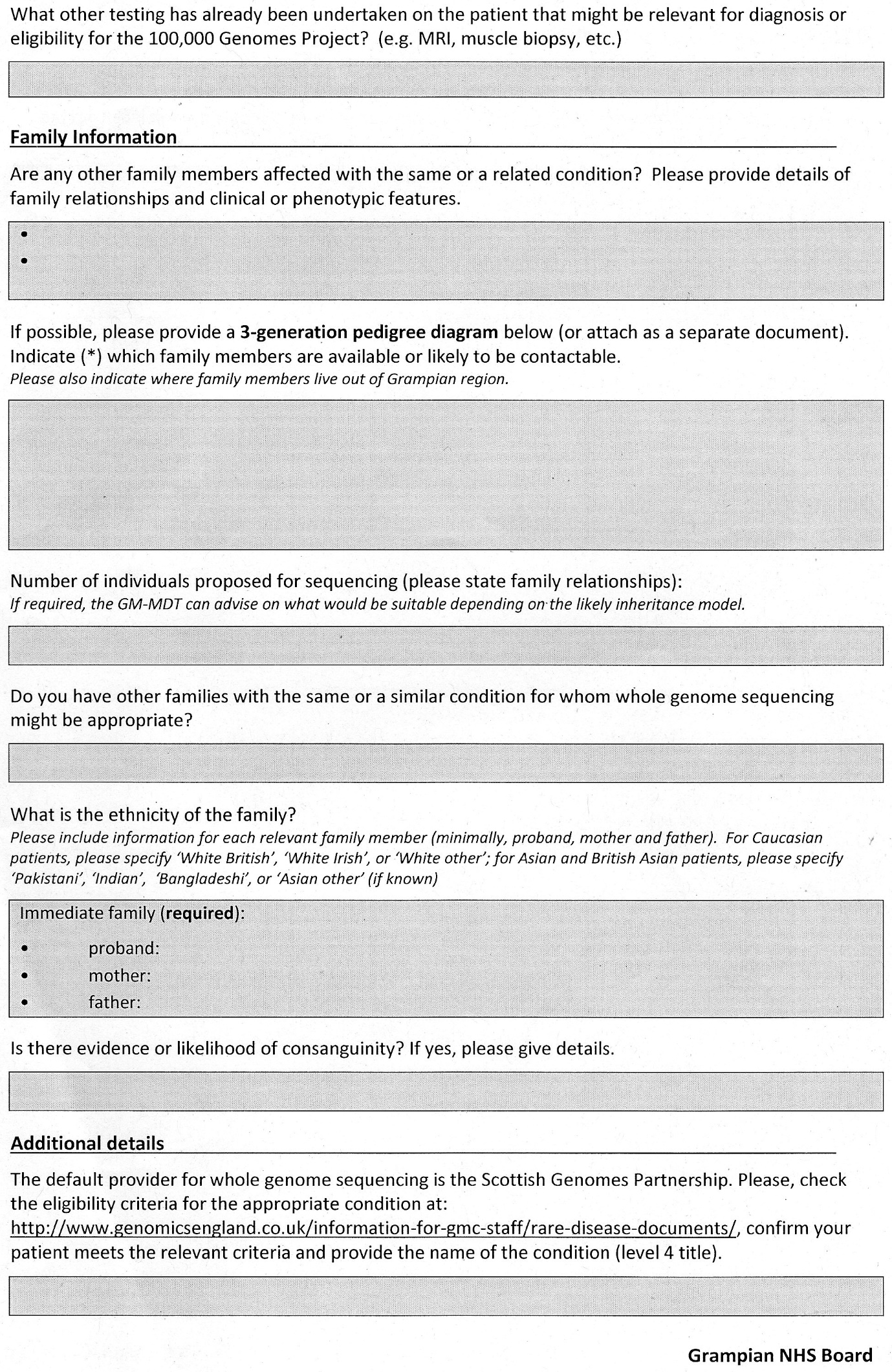


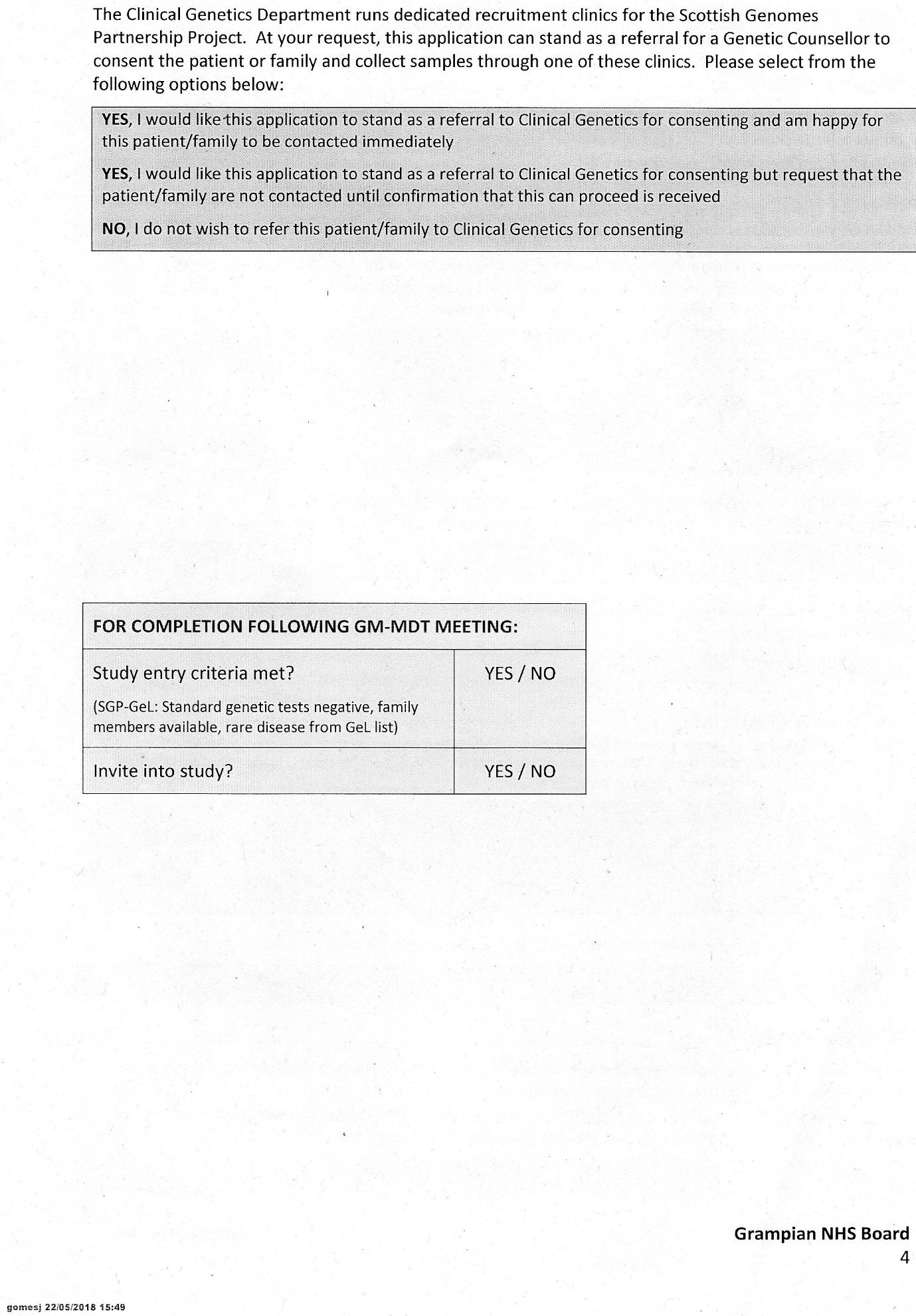

Supplement: Supplementary file 1 — Supplementary file1 (DOCX 3723 KB) [file 12687_2021_541_MOESM1_ESM.docx]
