# Supplementary material for: Continuing the sequence? Towards an economic evaluation of whole genome sequencing for the diagnosis of rare diseases in Scotland
Source: J Community Genet. 2021 Aug 20;13(5):487–501. doi: 10.1007/s12687-021-00541-4 (PMC9530076; doi:10.1007/s12687-021-00541-4)
Supplement: Supplementary file 2 — Supplementary file2 (DOCX 23 KB) [file 12687_2021_541_MOESM2_ESM.docx]

**Online Resource 2:** Sources of unit cost information for single gene, gene panel and epigenetic tests

|  | Name of gene or genetic test | | | | | | |
| --- | --- | --- | --- | --- | --- | --- | --- |
| Information Source for Costing |  | | | | | | |
| *NHS or UK testing laboratory* |  | | | | | | |
| Aberdeen | BRCA1/BRCA2 | GJB1 (aka CX32) | Fragile X | PMP22 |  |  |  |
| Belfast | CBS |  |  |  |  |  |  |
| Birmingham | ALMS1 | CHRNG | MLH1 | MSH2 | MSH6 | MLH1, MSH2 and 6 | |
|  | MTATP6 | ATP7A | COL4A5 | SCA 1,2,3,6,7 |  |  |  |
| Bristol | ANKRD11 | LITAF | MFN2 | NEFL |  |  |  |
| Cambridge | FTL | MAPT | SLC16A2 | COL11A1 | COL2A1 | MED12 | Nemo (IKBKG) |
| Cardiff | PARK7 | ACTG1/ACTB (actin) | DM1 | DM2 | OPA1 | TCF4 | VCAN |
| Dundee | CACNA1S | Mitochondrial cytopathy (3 genes) |  |  |  |  |  |
| Edinburgh | Smith-Lemli-Opitz | Spinal Muscular Atrophy |  |  |  |  |  |
| Exeter | LMNA | RET | SLC5A1 | ZIC3 |  |  |  |
| Glasgow | Angelman Syndrome | SLC2A1 | CDH7 | SLC2A1 | FKRP | MECP2 | Russell-Silver |
|  | SCN1A | Prader-Willi | Myotonic dystrophy | |  |  |  |
| Great Ormond Street Hospital | SMARCA2 | FGFR2 | FGFR3 | GNAS1 | ACADM | MYH7 | NPHS2 |
| Liverpool | SGCE | FXN |  |  |  |  |  |
| London Institute of Neurology | MTTL1 |  |  |  |  |  |  |
| London North West | GFAP |  |  |  |  |  |  |
| London South East | KAT6B | SEPN1 (SELENON) | SLC52A2 | SLC52A3 | SOD1 |  |  |
| London South West | ACTA2 | FBN1 | SMAD3 |  |  |  |  |
| Manchester | ORF15 (RPGR) | GBA | NF2 |  |  |  |  |
| Newcastle | ATN1 | GPC3 | NIPBL |  |  |  |  |
| Oxford | CHAT | CHRNE | EFNB1 | FLNA | FGFR3 | RAPSN (RAPSYN) | REN/UMOD |
|  | TCF12 | TCOF1 | UPD 14 (Imprinting disease), | 11p15 methylation | |  |  |
| Salisbury | P0 (MPZ) | ZFHX1B (ZEB2) |  |  |  |  |  |
| Sheffield | ATP7B | GCH1 | ATL1 | COL5A1/COL5A2 | COL6A1/2/3 | CPT2 | FH(HLRCC) |
|  | KCNA1 | AR | NBAS | PKD2 | SPAST |  |  |
| Other |  |  |  |  |  |  |  |
| *UK Genetic Testing Network* | ASPM (MCPH5) | DHCR7 | LMNA | MADD | Notch3 | NSD1 | PMP22 |
|  | POLG (POLG1) | PTEN | TPM2 |  |  |  |  |
| *Estimate based on numbers of amplicons ^1^* | AARS | ACTA1 | ACVR2B | AFG3L2 | ANO3 | ARID1B | ARSE (arylsufatase E) |
|  |  |  |  |  |  |  |  |
|  | ATM | ATP1A3 | ATRX | C9orf72 | CACNA1A | CACNB4 | CAPN3 |
|  | CAPS | CCDC78 | CDKL5 | CFAP53 | CFC1 | CFL2 | CLCN1 |
|  | COL11A2 | COL4A3 | COL4A4 | COL4A6 | CRELD1 | CYP27A1 | DCC |
|  | DCTN1 | DDHD1 | DHD7 | DIS3L2 | DNAJB6 | DNM2 | DYT1 |
|  | ECEL1 | EGR1 | EGR2 | EP300 | ERF | FA2H | FGFR1 |
|  | FHL1 | FMF (MEFV) | FOXC2 | FSHD (FRG1) | Fukutin (FKTN) | GAA | GDAP1 |
|  | GDF1 | GJB2 | GJB6 | GNAL | GPC6 | HLXB9 (MNX1) | HSPB1 |
|  | HSPB3 | HSPB8 | IFITM5 | IGHMBP2 | KBTBD13 | KCNJ2 | KLH40 |
|  | KLH41 | KLHL9 | LAMA2(65exons) | | LMOD3 | LZTFL1 | MID1 |
|  | MP2 (PMP2) | MTATP8 | MVK | MVKd | MYBPC1 | MYC1 | MYH11 |
|  | MYH3 | MYH8 | MYHFR | MYO18B | NEB | NEO2B | NFIX |
|  | NLRP12 | NOD2 | NODAL | NPHP4 | ORAI1(STIM1) | OTX2 | PAFAH1B1(WS1) |
|  | PANK2 | PEO1(TWNK) | PIEZO2 | PLP (PNPO) | PNKD | POLG2 | POMT1 |
|  | POMT2 | PREPL | PRKCG | PTCH1 | pyruvate dehydrogenase (PDP1) | | RASA1 |
|  | RRM2B | RYR1 | Sarcoglycan (SGCA) | | SBDS | SCN5A | SDHC |
|  | SETX | SLC1A3 | SLC25A4 | SLC5A7 | SMN (SMN1) | SNCA | SOX9 |
|  | SPG11 | SPG7 | SPR | SRD5A2 | STAC3 | STAT-3 | TH |
|  | THAP1 | Titin (TCAP) | TK2 | TNFRSF1A (TRAPS) | TNNT1 | TNNT2 | TNNT3 |
|  | TP53/p53 | TP63 | TPM3 | TTN | TUBB3 | UBE3A | UPF3B |
|  | VEGF (KDR) | VPS33B | WDR45 | WFS1 | YARS | ZBTB20 | ZC4H2 |
|  | ZDHHC9 |  |  |  |  |  |  |

^1^ where a unit cost value could not be obtained directly a method of costing was based on tests known test costs and the number of amplicons in the gene (Norbury et al., 2016), a single amplicon being around or under 500bp. For mutation screening the number of amplicons determines the score and this score is linked to the test cost.
