# Supplementary material for: Continuing the sequence? Towards an economic evaluation of whole genome sequencing for the diagnosis of rare diseases in Scotland
Source: J Community Genet. 2021 Aug 20;13(5):487–501. doi: 10.1007/s12687-021-00541-4 (PMC9530076; doi:10.1007/s12687-021-00541-4)
Supplement: Supplementary file 3 — Supplementary file3 (DOCX 97 KB) [file 12687_2021_541_MOESM3_ESM.docx]

**Online Resource 3:**

1. Interview schedule for qualitative think aloud interviews with SGP study participants.
2. Think Aloud interview instructions for the interviewer
3. **Interview Schedule for Affected Adults**

# Scottish Participation in the 100,000 Genomes Project

**What is important to you about whole genome sequencing?**

You have decided to take part in the Scottish Genomic Partnership (SGP) Research Study because you have a rare condition for which no clear cause has yet been found. By answering this questionnaire, you will help NHS Scotland to understand better how much people like you value whole genome sequencing as a genetic test for rare conditions. This will help NHS Scotland to decide how they might provide this test in the future.

The questionnaire is divided into four sections. **Section I** asks background questions about **you** and the rare condition. **Section II** is about **your health**. **Section III and IV** are concerned with understanding how much you value whole genome sequencing.

If you are not sure or prefer not to answer any question, please leave it blank. There are no right or wrong answers to the questions in this survey; we are interested in **your** opinion. All your answers will be **strictly confidential** and will not be used in any way you could be identified.

Once you have finished answering the questions, please ***hand your questionnaire back to the research staff before you leave the clinic.***

**Thank you very much for your participation!**

**Section I ABOUT YOU**

1. **What is your year of birth?** ___________
2. **What is your current marital status?**

*Please tick one box*

| Single | ❑ |
| --- | --- |
| Married/cohabiting | ❑ |
| Widowed / separated / divorced | ❑ |

1. **What is the highest level of education that you have completed?**

*Please tick one box*

| Primary school | ❑ |
| --- | --- |
| Secondary school | ❑ |
| Other professional/technical qualification after leaving school | ❑ |
| University degree or higher | ❑ |
|  |  |

1. **What is your main employment status?**

*Please tick one box only.*

Employed full-time ❑ Unemployed ❑

Employed part-time ❑ Retired ❑

Student full-time ❑ Student part-time ❑

Homemaker/carer ❑ Self-employed ❑

Other (please specify) ___________________________

1. **How many people live in the same house as you including you?**

*Please provide one number per row*

| Aged 15 or under | _____________ |
| --- | --- |
| Aged 16 or over | _____________ |

1. **How much is your monthly household income from all sources (before tax)? (Include benefits or pensions as well as income from wages etc.)**

*Please tick one box*

| Up to £1,000 per month | ❑ | £4,000 and up to £5,000 per month | ❑ |
| --- | --- | --- | --- |
| £1,000 and up to £2000 per month | ❑ | £5,000 and above per month | ❑ |
| £2,000 and up to £3000 per month | ❑ |  |  |
| £3,000 and up to £4,000 per month | ❑ |  |  |

**Section II YOUR HEALTH**

In this section, we are interested to find out more about **your** health.

| 1. **By placing a tick in one box in each group below, please indicate which statements best describe your own health state today.** | | |
| --- | --- | --- |
|  | |  |
| **Mobility** | |  |
| I have no problems in walking about | | ❑ |
| I have some problems in walking about | | ❑ |
| I am confined to bed | | ❑ |
|  | |  |
| **Self-Care** | |  |
| I have no problems with self-care | | ❑ |
| I have some problems washing or dressing myself | | ❑ |
| I am unable to wash or dress myself | | ❑ |
|  | |  |
| **Usual Activities** *(e.g. work, study, housework, family or leisure activities)* | |  |
| I have no problems with performing my usual activities | | ❑ |
| I have some problems with performing my usual activities | | ❑ |
| I am unable to perform my usual activities | | ❑ |
|  | |  |
| **Pain / Discomfort** | |  |
| I have no pain or discomfort | | ❑ |
| I have moderate pain or discomfort | | ❑ |
| I have extreme pain or discomfort | | ❑ |
|  | |  |
| **Anxiety / Depression** | |  |
| I am not anxious or depressed | | ❑ |
| I am moderately anxious or depressed | | ❑ |
| I am extremely anxious or depressed | | ❑ |
|  |  |  |
|  |  |  |
|  |  |  |
|  |  |  |
| 1. **To help people say how good or bad a health state is, we have drawn a scale (rather like a thermometer) on which the best state you can imagine is marked 100 and the worst state you can imagine is marked 0**. |  |  |
|  |  |  |
| We would like you to indicate on this scale how good or bad your own health is **today**, in your opinion. Please do this by drawing a line from the box below to whichever point on the scale indicates how good or bad your health state is today. |  |  |
|  |  |  |
|  |  |  |

Best imaginable health state

Your own health state today

Worst imaginable health state

10

0

20

30

40

50

60

80

70

90

100

5

15

25

35

45

55

75

65

85

95

1. **If you would like to add anything else related to other day-to-day living problems you suffer from, please write down here.**
2. **How old were you when early signs symptoms started to show due to the rare condition?**

___________ Years/Months (cross out as appropriate)

Unsure/ Don’t know ❑

1. **How long have you been trying to find a genetic cause for this condition?**

___________ Years/Months (cross out as appropriate)

Unsure/ Don’t know ❑

1. **What tests have been done to try to diagnose your condition?**
   *Please tick all that apply*

| Other genetic tests | ❑ |
| --- | --- |
| Operative procedure (biopsies, surgeries) | ❑ |
| Other non-genetic tests (e.g. MRI, CT, x-ray) | ❑ |
| Unsure  / Don’t know | ❑ |

1. **Do you have any children of your own?**

| Yes | ❑ |
| --- | --- |
| No | ❑ |

**Section III WHAT IS IMPORTANT TO YOU ABOUT WHOLE GENOME SEQUENCING?**

These are some key things to remember about whole genome sequencing:

- Some patients with rare diseases may get a genetic diagnosis for their rare disease for the first time, but many will not. This can vary depending on the type of condition.
- Your relative will usually wait from **6 months to 2 years** to know whether or not a genetic cause for their rare condition has been found.
- **Health-related information** will be provided to other family members if a genetic cause is found to affect them directly, this means that they will be contacted if they are at risk of **developing** and/or **passing** on the rare condition.
- You can choose whether or not you want to get **additional information** about other health conditions that whole genome sequencing might reveal.
- This research will benefit **future healthcare** for **other people** with similar rare genetic conditions.

1. **On the previous page we described five key features of whole genome sequencing. Please tell us how important the following different aspects of whole genome sequencing are to you.**

**Please tick only one box after each statement below.**

| **Knowing that some people who have this test will get a diagnosis for their rare condition, but many will not** | | | | |
| --- | --- | --- | --- | --- |
| Not at all important | Slightly important | Moderately Important | Very Important | Extremely important |
| ❑ | ❑ | ❑ | ❑ | ❑ |
|  |  |  |  |  |
|  |  |  |  |  |
| **The test results can take from 6 months to 2 years** | | | | |
| Not at all important | Slightly important | Moderately Important | Very Important | Extremely important |
| ❑ | ❑ | ❑ | ❑ | ❑ |
|  |  |  |  |  |
|  |  |  |  |  |
| **The test may let other family members know if they are at risk of developing and passing on the rare condition** | | | | |
| Not at all important | Slightly important | Moderately Important | Very Important | Extremely important |
| ❑ | ❑ | ❑ | ❑ | ❑ |
|  |  |  |  |  |
|  |  |  |  |  |
| **You can choose to get any available additional information from the test about other health-related conditions that may affect other people in your family** | | | | |
| Not at all important | Slightly important | Moderately Important | Very Important | Extremely important |
| ❑ | ❑ | ❑ | ❑ | ❑ |
|  |  |  |  |  |
|  |  |  |  |  |
| **This research will benefit future healthcare for people with rare genetic conditions** | | | | |
| Not at all important | Slightly important | Moderately Important | Very Important | Extremely important |
| ❑ | ❑ | ❑ | ❑ | ❑ |

1. **If there are any other benefits you hope to gain by participating in this study, please write them down here.**

1. **If there any disadvantages you see of this study, please write them down here.**

**Section V WHAT DO YOU VALUE ABOUT WHOLE GENOME SEQUENCING**

In this section we interested in how much you value whole genome sequencing. One way of doing this is to ask you how much you are willing to pay for the test.

1. **Thinking about the previous section, please answer the following.**

- **Right now, whole genome sequencing is not yet available as a free routine test in NHS Scotland and was only available free to you as a part of our research study.**
- **Please imagine that you would have to pay privately if you wished to have the whole genome sequencing test.**
- **How much would you be prepared to pay for the whole genome sequencing test?**

*Remember, any money you spend on whole genome sequencing cannot be spent on other things (including household bills, childcare costs, other healthcare costs, travel, leisure activities etc.).*

**The maximum amount I would be willing to pay for whole genome sequencing is:**

**£___________**

1. **If you said £0.00, please tell us why you are not prepared to pay for whole genome sequencing?**

| **THANK YOU FOR COMPLETING THE QUESTIONNAIRE!** |
| --- |

| For official use only: | |
| --- | --- |
| GeL ID |  |
| SGP ID |  |
| Family ID |  |

**2) Think aloud interview instructions**

These instructions are to be followed during the SGP study optional questionnaire development session visit described in SGP:HERU SOP 1.

1. **INTRODUCTION**

Hello, my name is Blanca Moran, and I am part of the research team in the SGP study at the University of Aberdeen. I would like to **thank you** for coming here to help us out at this *optional* questionnaire development session.

- We are here **to test** a questionnaire that will be used in a **later study** to understand the benefits of whole genome sequencing (WGS).
- We are interested in finding out if the **questionnaire is easy to understand**, and if we are **asking** the **right** questions in the **right** way.
- One way to find out what you are thinking about when you answer the questionnaire is to ask you to **think out loud** as though you were talking to yourself as you answer the questions.

***Do you have any other questions before we proceed?***

1. **PARTICIPANT CONSENT FORM AND RECORDING**

***Explaining the consent form.***

- This consent form has two boxes, in the first part of the consent form; you are letting us know that you decided to take part in this optional session and that you can withdraw at any time. You will validate this part by writing your initials at the bottom of the box.
- In the second part of the consent form we tell you how your data will be treated confidentially and will only be accessed by people within the SGP study, we will need you to put your initials at the bottom of the box to know you agree.
- Finally, you will need to write your name, put your DOB, sign it, and put today’s date.

I will write my name, sign, and date the form that I have received. *Your genetics consultant will send you a copy of the consent form by post.*

- I would also like to let you know that from the moment I give you the questionnaire **I will record** what you say during the session and type up your comments afterwards**, this is to make it easier for me to remember what you are saying**. I will stop the recording once we finish with the questionnaire. The recording and questionnaire will be identified using your study identifiers, not your name.

***Is there anything you want to ask before I hand out the questionnaire?***

1. **EXPLAIN PROCEDURE**

Here is a copy of the questionnaire.

Remember there are **no** right or wrong answers; we are **testing the questionnaire** not you.

At any point, if there is anything unclear or misleading just tell me. When you are answering the questions, talk out loud to tell me what you are thinking as you decide which answer to choose and why you are choosing it. I won’t be able to help you to answer the questions but if you need anything explained, ***I’ll try to help at the end of the questionnaire***.

Tell us you reaction to everything, the questionnaire, the way it looks, anything that seems strange or you don’t understand.

***If they have any clinical questions (say this if they ask about anything clinical):***

Also, please keep in mind that I am here as a part of the research team for the questionnaire session only, if you have any questions regarding any clinical issue you will need to discuss that with your genetics counsellor.

***Probes that might be used during the questionnaire session:***

- What are you thinking right now?
- Remember to read aloud for me—it’s up to you what you read, but whatever you decide to read please do aloud so I know what you are looking at.
- Can you tell me more about that?
- Could you describe that for me?
- Don’t forget to tell me what you are thinking as you do that.

1. **RECORD RELEVANT COMMENTS, ERRORS, HESITATIONS, AND OTHER INDICATORS OF POTENTIAL PROBLEMS DURING COMPLETION (TO BE USED TO FRAME FOLLOW-UP QUESTIONS).**

- Did respondent read the instructions, side notes, etc.?
- What reactions did respondent volunteer, if any?
- Did respondent read the instructions?
- Any reactions/hesitations/questions to the roster instructions?

1. **AFTER THE QUESTIONNAIRE - POSSIBLE PROBES**

- Overall how easy / difficult have you found this.
- Did you find that it got easier as you went through the questions?
- Were some easier / more difficult than others?
- Can you remember which ones?
- Were the instructions clear?
- If you found it unclear do you have any ideas, how we can make it clearer?
- If this questionnaire arrived at your residence in the post, how soon do you think would you respond?
